# The Family-Home Nutrition Environment and Dietary Intake in Rural Children

**DOI:** 10.3390/nu7125495

**Published:** 2015-11-25

**Authors:** Jennifer A. Jackson, Ellen Smit, Melinda M. Manore, Deborah John, Katherine Gunter

**Affiliations:** School of Biological and Population Health Sciences, Oregon State University, Corvallis, OR 97331, USA; ellen.smit@oregonstate.edu (E.S.); melinda.manore@oregonstate.edu (M.M.M.); deborah.john@oregonstate.edu (D.J.); kathy.gunter@oregonstate.edu (K.G.)

**Keywords:** nutrition, diet, food insecurity, family, children, overweight and obesity, rural

## Abstract

Obesity and food insecurity rates are higher among rural compared to non-rural populations. Little is known, however, about how family-home environments influence childhood obesity-related behaviors, particularly in rural settings. This study examined associations between the family-home nutrition (FN) environment, food insecurity, and dietary intake (fruits, vegetables, whole grains, dairy, protein foods, and added sugars) in rural elementary school-age children (grades K-5/6; *n* = 102). Parents/caregivers completed surveys on FN, food insecurity, and the Block Kids Food Screener (BKFS). Body mass index (BMI, kg/m^2^) was calculated from measured height and weight. Approximately 33% of children were classified as overweight/obese and 28% of families were at-risk for food insecurity. Multivariable linear regression analyses examined associations between dietary intakes with FN and food insecurity. More favorable FN scores were associated with lower added sugar intake (B = −1.38, *p* = 0.04) and higher vegetable (B = 0.15, *p* < 0.001), fruit (B = 0.71, *p* = 0.01), and dairy (B = 0.31, *p* < 0.001) intakes. No significant associations were found between food insecurity and dietary intake. Given the association between higher FN scores and more favorable dietary intake, promoting healthy FN environments among rural children is warranted.

## 1. Introduction

Prevention of childhood obesity is a public health priority in the United States (US) [1,2], with population disparities presenting unique challenges for prevention efforts [3,4,5,6]. One such disparity is the higher prevalence of obesity among rural compared to non-rural children [7,8,9,10,11]. The most current available evidence indicates that rural children have 26% greater odds of obesity compared to urban children [11], yet evidence to explain this disparity is inconclusive.

Behavioral and environmental factors in the family-home, such as those related to healthy eating, may influence children’s risk for obesity [12,13]. Among non-rural populations, evidence suggests associations between children’s eating behaviors and family-level factors including parent education and role modeling [14,15,16,17,18], family food rules [14,19], and family meal patterns [20,21,22,23]. Additionally, other home environmental factors, such as availability of healthy foods [14,18,24], eating while watching TV [25], and fast food consumption [15,25], may make it easier or harder for children to eat healthfully. Unfortunately, research examining these and other obesity-promoting or -preventing factors in rural family-home settings is limited.

Few studies have directly compared determinants of obesity between urban and rural children, and results are conflicting [11]. For example, while two studies reported that rural children were more likely to be obese than urban children, one found no significant differences in dietary intake or physical activity between rural and urban children, while the other study showed that compared to urban children, rural children consumed more calories and reported participating in exercise more often [7,10]. These findings suggest that environmental factors may contribute to childhood obesity risk in rural areas. Understanding whether and how rural family-home environments may influence obesity-related behaviors will inform public health strategies to promote weight-health among rural children.

Rural populations also experience higher rates of food insecurity compared with non-rural populations [26,27]. Although food insecurity and obesity often coexist, evidence for an association between these factors in children is inconsistent [28,29,30]. Furthermore, some research suggests that dietary behaviors differ between food-insecure and food-secure children [31], whereas others have found no differences [32]. Given that obesity and food insecurity rates are higher among rural populations, it is important to understand lifestyle factors that potentially contribute to this association.

The first aim of this cross-sectional study was to determine if family-home nutrition (FN) factors are associated with dietary intake (*i.e.*, food groups and added sugars) in rural children. The second aim was to determine if food insecurity is associated with dietary intake in rural children. It was hypothesized that more favorable FN factors would be associated with healthier dietary intake and that being at-risk for food insecurity would be associated with less healthy dietary intake.

## 2. Methods

### 2.1. Participants

Data were collected in the context of a larger childhood obesity prevention study, Generating Rural Options for Weight (GROW) Healthy Kids and Communities [33]. GROW study sites included six communities (population <10,000), designated as rural by the US Census [34], located within three geographically diverse Oregon counties. Eligible elementary schools (≥50% of families eligible for federal school meal programs) within each community were randomly selected to participate in GROW (*n* = 6 schools located in 6 different communities).

All families with elementary school-age children attending GROW elementary schools (grades K-5/6; *N* = 2200 children) were eligible to participate in the study. Participants were recruited via an informational packet sent from the school to the home. Recruitment materials included a brief description of the study, the steps necessary to enroll, informed consent documents, survey instruments, and a postage-paid envelope. Families could enroll by returning the forms included in the packet or by completing the documents online. Approximately 12% of children (*n* = 270) and their parents/caregivers consented to participate in the GROW study between 2012 and 2014. The Oregon State University Institutional Review Board approved all protocol and procedures prior to initiation of this study.

### 2.2. Data Collection and Measures

Data for the present study included survey responses about children’s dietary intake, FN factors, family food insecurity, and parent and child demographics. Parents/caregivers (henceforth referred to as parents) completed all surveys via print or electronic format between summer and fall of 2014. Children’s height and weight were measured during fall of 2014 and used to calculate body mass index (BMI, kg/m^2^). Details on the survey instruments and BMI measures are provided below.

#### 2.2.1. Dietary Intake

The Block Kids Food Screener (BKFS) [35] was used to assess children’s dietary intake; specifically, food groups and added sugars. Relative validity of the BKFS was examined in a sample of youth aged 10–17 years (*n* = 99), using three 24-h dietary recalls as the reference measure [36]. The de-attenuated correlations between estimates produced by the two dietary assessment methods ranged from 0.478 to 0.878. Although the BKFS is a validated measure of estimated food group and added sugar intakes, it has not been validated for total energy intake.

The two page BKFS survey consists of 41 items. Parents reported the frequency and quantity of foods and beverages consumed by their child during the previous week. The BKFS food list was developed from National Health and Nutrition Examination Survey (NHANES) data for youth aged 2–17 years. Portion sizes are assigned according to the age and sex of the child based on amounts consumed in the most recent NHANES survey 24-h recall data.

BKFS summary data were provided by NutritionQuest [35] and included the following estimated average daily intakes: ounce/cup equivalents of fruits, vegetables, whole grains, dairy, protein foods (meat, fish, and poultry); added sugar (tsp); and total energy (kcals). Food group servings were defined by the US Department of Agriculture (USDA) MyPyramid Equivalents Database (MPED) 2.0 [37]. For each MyPyramid food group, the MPED provides the number of MyPyramid equivalents that are present in 100 g of each of the foods consumed by participants in NHANES. Recommended intakes based on the 2010 Dietary Guidelines for Americans were used for comparison [38].

For the purpose of this study, estimated total energy intake was used to standardize dietary intake (*i.e.*, food groups and added sugars) per 1000 kcals **(4128 kJ)** and to identify over/under-reporters. No standard exclusion criteria exist for identifying over/under-reporting of dietary intake estimated by food screening instruments. Therefore, we used the method described by Choumenkovitch and colleagues [39] to define over-reporting as estimated total energy intake >5000 kcal/day and under-reporting as two or fewer foods reported per day.

#### 2.2.2. Family-Home Nutrition (FN) Factors

Parents completed the Family Nutrition and Physical Activity (FNPA) screening tool, a previously validated instrument designed to assess evidence-based family environmental and behavioral factors that predispose young children to becoming overweight [12,13]. Ihmels and colleagues demonstrated internal consistency of the FNPA instrument (α = 0.72) and found that low FNPA scores were significantly associated with child overweight status (≥85th BMI-for-age percentile) [12,13].

The FNPA instrument includes 20 items in two component areas (nutrition and physical activity) (Table 1). Each component contains five domains (e.g., Meal Patterns) defined by two items each (e.g., My child eats breakfast + Our family eats meals together).

**Table 1:** Family Nutrition and Physical Activity (FNPA) factors [12].

Nutrition Component
**Meal Patterns**
FNPA 1: My child eats breakfast
FNPA 2: Our family eats meals together
**Eating Habits**
FNPA 3: Our family eats while watching TV
FNPA 4: Our family eats fast food
**Food Choices**
FNPA 5: Our family uses microwave or ready to eat foods
FNPA 6: My child eats fruits and vegetables at meals or snacks
**Beverage Choices**
FNPA 7: My child drinks soda pop or sugar drinks
FNPA 8: My child drinks low fat milk at meals or snacks
**Restriction/Reward**
FNPA 9: Our family monitors eating of chips, cookies, and candy
FNPA 10: Our family uses candy as a reward for good behavior
**Physical Activity Component**
**Screen Time Behavior/Monitoring**
FNPA 11: My child spends less than 2 h on TV/games/computer per day
FNPA 12: Our family limits the amount of TV our child watches
**Healthy Environment**
FNPA 13: Our family allows our child to watch TV in their bedroom
FNPA 14: Our family provides opportunities for physical activity
**Family Activity Involvement**
FNPA 15: Our family encourages our child to be active every day
FNPA 16: Our family finds ways to be physically active together
**Child Activity Involvement**
FNPA 17: My child does physical activity during his/her free time
FNPA 18: My child is enrolled in sports or activities with a coach or leader
**Family Routine**
FNPA 19: Our family has a daily routine for our child’s bedtime
FNPA 20: My child gets 9 h of sleep a night

All items coded on a 4-pt scale (1, almost never; 2, sometimes; 3, usually; 4, almost always); items 3, 4, 5, 7, 10, and 13 are reverse coded.

Item response categories were coded on a four-point scale as “Almost Never” (1); “Sometimes” (2); “Usually” (3); and “Almost Always” (4). All items were coded such that higher scores indicated more favorable behaviors and environments. For example, a high score in the *Restriction*/*Reward* domain reflects a family who “almost always” monitors eating of chips, cookies, and candy and “almost never” uses candy as a reward for good behavior. Previous research suggests that a higher total FNPA score reflects more favorable family policies and practices, inferring lower risk for child overweight [13]. For this study, we examined the FN component of the FNPA, including the nutrition domains and individual items, in association with dietary intake. We further examined the association between the total FNPA score and dietary intake.

#### 2.2.3. At-risk for Food Insecurity

To assess whether a family was “at-risk for food insecurity”, parents completed a validated 2-item food insecurity screening instrument [40]. This instrument has been found to have high sensitivity (97%), good specificity (83%), and convergent validity among a large population (*n* = 30,098) of low-income families with young children. The food insecurity screener includes the following two statements to which individuals are asked to respond: (1) “Within the past 12 months, we worried if our food would run out before we got money to buy more”; and (2) “Within the past 12 months, the food we bought just didn’t last and we didn’t have money to get more”. Item response categories were “never true”, “sometimes true”, and “often true”. Responses were dichotomized for analysis (often true/sometimes true *versus* never true) and a “yes” response to either statement was used to identify families at-risk for food insecurity.

#### 2.2.4. Body Mass Index (BMI)

Height and weight measurements were obtained by trained research staff. Height was measured to the nearest 1 mm using a portable stadiometer. Weight was measured to the nearest 0.1 kg using a digital scale. Measurements were repeated three times on each child and the averages were used to calculate BMI (kg/m^2^).

Children were classified as underweight (<5th percentile), normal weight (5th to <85th percentile), overweight (85th to <95th percentile), or obese (≥95th percentile) according to the sex-specific Centers for Disease Control and Prevention (CDC) BMI-for-age growth charts [41,42,43]. BMI scores were converted to z-scores using the sex- and age-specific parameters from the CDC growth charts.

#### 2.2.5. Covariates

Family demographics included eligibility for free- or reduced-cost school meals (yes, no) and parent education level (never attended school, grades 1–8, grades 9–11, grade 12 or GED, 1–3 years college, or 4 years or more college; recoded for analyses as: grade 12 or less, 1–3 years college, 4 years or more college). Child-level variables were age (years), sex (female, male), race (American Indian or Alaska Native, Asian, African American, Native Hawaiian or Other Pacific Islander, White), and ethnicity (Hispanic or Latino, Non-Hispanic or -Latino; recoded for analyses as Race/Ethnicity: non-Hispanic or -Latino white, Other). School (*n* = 6) and BMI (standardized for sex and age) were also considered as potential covariates.

### 2.3. Data Analysis

We received BKFS surveys from the parents of 38% (*n* = 102) of the 270 children enrolled in the GROW family study. No children were excluded based on over/under-reporting criteria. Two children were excluded due to missing data on one or more primary variables (*i.e.*, BKFS, FNPA, or food insecurity), and five were excluded for missing data on one or more of the covariates used for analyses. The final sample size was 95.

Dietary intake data were analyzed both as absolute amounts of food groups (cups or grams/day) and added sugar (grams/day) consumed and as food density consumed (servings/1000 kcal (4128 kJ)). Estimated Energy Requirements (EER) were calculated for all children with available height and weight data (*n* = 82) based on the Dietary Reference Intakes (DRI) sedentary activity levels for children (e.g., Physical Activity Level (PAL) = 1.0) [44]. Descriptive statistics were examined for all variables. Unadjusted linear regression models were used to examine associations between dietary variables with FNPA and food insecurity. Associations between BMI and FNPA, and BMI and food insecurity were examined using linear and logistic regression models. Bivariate associations were examined between all covariates and dependent variables. Due to small cell sizes, some demographic variable categories (e.g., race/ethnicity, parent education level) were collapsed or dichotomized for analysis. Covariates significant at the level of *p* < 0.1 were retained for adjusted models.

Associations were then examined using multivariable regression models, adjusted for the retained covariates. The Stata (version 13, 2013, StataCorp, College Station, TX, USA) cluster option was used to account for potentially correlated observations within families. Two-way interactions between independent variables and significant covariates were examined. Akaike’s information criterion was used for model comparisons. Residual plots and normality tests for residual distributions were used to assess model assumptions and goodness of fit. For final models, statistical significance was originally set at α = 0.05. False Discovery Rate (FDR) adjusted p-values for multiple comparisons were also computed [45]. Data analyses were performed using Stata and R (version 3.1, 2015, R Foundation for Statistical Computing, Vienna, Austria).

## 3. Results

### 3.1. Study Participants

Sample characteristics are displayed in Table 2. Total FNPA and FN scores were generally high, with average scores of 3.3 (on a scale from 1 to 4). The median number of children per family was 2. Of the 82 children with measured BMI data, approximately 33% of children were classified as overweight or obese. Compared to the larger population across the six GROW schools, this sample was not significantly different based on BMI percentile (*p* > 0.50), but included a significantly lower percentage of children eligible for free or reduced school meals (47% *vs.* 68%) and higher percentage of white children (93% *vs.* 75%) (*p* < 0.0001) (data not shown).

**Table 2:** Characteristics of rural elementary school-age children.

Characteristic	*n*	Mean (SD) or %
**Age (years)**, mean	102	8.4 (2.0)
**Sex**, %	102	
Female	47	46.1
**Race/Ethnicity**, %	95	
Non-Hispanic or -Latino white	84	88.4
**Parent education**, %	102	
Grade 12 or less	11	10.8
1–3 years of college	45	44.1
4 or more years of college	46	45.1
**Eligible for free/reduced school meals**, %	101	
Yes	47	46.5
**At-risk for food insecurity**, %	100	
Yes	28	28.0
**BMI**, mean	82	18.4 (4.0)
**BMI percentile**, mean	82	65.1 (27.7)
Underweight, %	2	2.4
Normal weight, %	53	64.6
Overweight, %	13	15.9
Obese, %	14	17.1
**BMI z-score**, mean	82	0.6 (1.1)
**FNPA score**, mean	100	
FNPA Total ^a^	100	3.3 (0.3)
FN ^b^	100	3.3 (0.3)

^a^ FNPA Total; average of 20 FNPA items coded on 4-pt scale (1, almost never; 2, sometimes; 3, usually; 4, almost always); ^b^ FN; average of 10 FNPA nutrition items. FNPA: Family Nutrition and Physical Activity; BMI: Body mass index; SD: Standard Deviation.

The sample mean EER was 1591 kcals/day (*n* = 82). Thus, we compared our sample mean dietary intakes from the BKFS with recommended average daily intake amounts for a 1600 kcal level, based on the 2010 Dietary Guidelines for Americans [38] (Table 3). Children in our study consumed significantly less than the recommended amounts of vegetables, whole grains, dairy, and protein foods, and significantly more than the recommendations for fruit and added sugars (*p* < 0.001).

**Table 3:** Dietary intakes from the Block Kids Last Week Food Screener (*n* = 95).

	Study Sample		Recommended ^a^
Dietary Intakes	Mean (SD)	Mean (SD) per 1000 kcals (4128 kJ)	per 1000 kcals (4128 kJ)
**Fruits (cups/day)**	1.63 (0.99)	1.34 (0.68)	0.94
**Vegetables (total)** ^b^ **(cups/day)**	0.98 (0.50)	0.84 (0.37)	1.25
**Whole Grains (g/day)**	20.00 (17.00)	17.00 (14.00)	53.00
**Dairy (cups/day)**	1.84 (0.91)	1.55 (0.65)	1.88
**Protein Foods (g/day)**	63.00 (39.00)	53.00 (22.00)	88.00
**Added Sugar (g/day)**	22.00 (12.00)	18.00 (9.00)	9.00
**Energy (kcal/day)**	1193 (410)	-	-
**(kJ/day)**	4926 (1691)	-	-

^a^ Recommended intake per 1000 kcals (4128 kJ) based on Dietary Guidelines for Americans 2010 recommended average daily intake amounts for 1600 kcal (6605 kJ) level [38]; ^b^ Includes potatoes and legumes. SD: Standard Deviation.

### 3.2. Family-Home Nutrition (FN) and Dietary Intake

Results show that children with a higher, more favorable FN component score tended to have lower intake of added sugar (B = −2.37, *p* = 0.06), driven in part by the Food Choices domain (B = −0.77, *p* = 0.01) (Table 4). Specifically, lower added sugar intake in children was significantly associated with families who used microwave or ready-to-eat foods less often (B = −1.38, *p* = 0.04). Children whose parents reported more frequent consumption of fruits and vegetables at meals or snacks also tended to consume less added sugar (B = −0.75, *p* = 0.05). In addition, if parents reported their children consumed sugar-sweetened beverages less often, then added sugar intake was significantly lower (B = −1.36, *p* = 0.04). Finally, a more favorable FNPA total score was associated with lower intake of added sugar (B = −2.47, *p* = 0.04).

A higher FNPA total score was also associated with higher vegetable intake (B = 0.29, *p* = 0.04). If parents reported that their children consumed more fruits and vegetables at meals or snacks, then vegetable intake was higher (B = 0.17, *p* = 0.01). Vegetable consumption also tended to be higher for children with families who used microwave or ready-to-eat foods less often and those who monitored consumption of chips, cookies, and candy (B = 0.20, 0.11; *p* = 0.06, 0.06).

A higher FN component score was positively associated with fruit intake (B = 0.71, *p* = 0.01), driven by the Meal Patterns and Food Choices domains (B = 0.20, 0.24; *p* = 0.01, 0.04, respectively). If parents reported that their children ate breakfast more often or that children more frequently consumed fruits and vegetables at meals or snacks, then fruit intake was higher (B = 0.24, 0.33; *p* = 0.04, <0.001, respectively). In addition, if parents reported that their families ate meals together more often, then the children had higher fruit intakes (B = 0.25; *p* = 0.04). Finally, if parents reported that their children frequently consumed low-fat milk at meals or snacks, then dairy intakes were higher (B = 0.31, *p* < 0.001). No significant associations were observed between FN or total FNPA and dietary intakes of whole grains or protein foods. We further examined and observed no associations between FN or total FNPA and BMI in a subgroup of children with complete data (*n* = 76; data not shown).

**Table 4:** Multivariable linear regression examining associations between FNPA factors and at-risk for food insecurity with dietary intakes (*n* = 95).

	Fruits (Cups /Day) per 1000 kcals (4128 kJ) ^a^		Vegetables (Cups /Day) per 1000 kcals (4128 kJ) ^b^		Whole Grains (g)/Day) per 1000 kcals (4128 kJ) ^c^		Dairy (Cups Day) per 1000 kcals (4128 kJ) ^d^		Protein Foods (g)/Day) per 1000 kcals (4128 kJ) ^c^		Added Sugar (g)/Day) per 1000 kcals (4128 kJ) ^d^	
**Variable**	B Coef	p-adj †	B Coef	p-adj	B Coef	p-adj	B Coef	p-adj	B Coef	p-adj	B Coef	p-adj
**FNPA Total**	0.58	0.06	0.29	0.04 *	0.01	0.95	0.14	0.78	−0.03	0.95	−2.47	0.04 *
**FN Component**	0.71	0.01 *	0.31	0.07	−0.08	0.78	0.20	0.72	0.15	0.81	−2.37	0.06
**Meal Patterns**	0.20	0.01 *	0.08	0.12	0.01	0.92	0.01	0.95	0.09	0.60	−0.55	0.26
FNPA 1: My child eats breakfast	0.24	0.04 *	0.10	0.19	0.13	0.14	−0.10	0.78	0.18	0.17	−0.61	0.53
FNPA 2: Our family eats meals together	0.25	0.04 *	0.10	0.17	−0.06	0.72	0.09	0.67	0.06	0.84	−0.77	0.32
**Eating Habits**	0.16	0.17	0.04	0.69	−0.05	0.70	−0.05	0.81	0.08	0.72	−0.55	0.22
FNPA 3: Our family eats while watching TV	0.18	0.17	0.01	0.95	0.00	0.97	−0.09	0.71	0.12	0.68	−0.63	0.31
FNPA 4: Our family eats fast food	0.26	0.44	0.12	0.54	−0.17	0.26	0.02	0.95	0.08	0.86	−0.82	0.28
**Food Choices**	0.24	0.04 *	0.15	0.00 ***	−0.02	0.84	−0.09	0.53	0.13	0.38	−0.77	0.01 *
FNPA 5: Our family uses microwave or ready to eat foods	0.12	0.70	0.20	0.06	−0.09	0.71	−0.09	0.80	0.16	0.72	−1.38	0.04 *
FNPA 6: My child eats fruits and vegetables at meals or snacks	0.33	0.00 ***	0.17	0.01 *	0.01	0.95	−0.12	0.53	0.14	0.52	−0.75	0.05
**Beverage Choices**	0.03	0.81	−0.03	0.67	−0.01	0.95	0.25	0.00 ***	−0.14	0.26	−0.06	0.90
FNPA 7: My child drinks soda pop or sugar drinks	0.30	0.17	0.11	0.37	0.20	0.17	−0.06	0.84	0.12	0.78	−1.36	0.04 *
FNPA 8: My child drinks low fat milk at meals or snacks	−0.03	0.84	−0.06	0.35	−0.06	0.60	0.31	0.00 ***	−0.20	0.17	0.23	0.60
**Restriction/Reward**	0.09	0.55	0.08	0.17	−0.03	0.78	0.02	0.86	0.02	0.94	−0.38	0.35
FNPA 9: Our family monitors eating of chips, cookies, candy	0.10	0.55	0.11	0.06	0.01	0.95	0.12	0.32	−0.01	0.95	−0.66	0.17
FNPA 10: Our family uses candy as a reward for good behavior	0.06	0.72	0.03	0.81	−0.09	0.60	−0.13	0.44	0.07	0.81	0.07	0.94
**At-risk for Food Insecurity**	0.18	0.55	0.04	0.86	−0.09	0.74	−0.08	0.84	0.19	0.69	−0.21	0.84

^a^ Clustered for multiple children in families and adjusted for child sex; ^b^ Clustered for multiple children in families and adjusted for child race/ethnicity, age, and school; ^c^ Clustered for multiple children in families; ^d^ Clustered for multiple children in families and adjusted for school; † FDR-adjusted *p*-value; * *p* < 0.05; ** *p* < 0.01; *** *p* < 0.001; FNPA Total; average of 20 items coded on 4-pt scale (1, almost never; 2, sometimes; 3, usually; 4, almost always); FN Component; average of 10 FNPA nutrition items; Meal Patterns; average of 2 items: My child eats breakfast + Our family eats meals together; Eating Habits; average of 2 items: Our family eats while watching TV + Our family eats fast food (both items reverse coded); Food Choices; average of 2 items: Our family uses microwave or ready to eat foods (reverse coded) + My child eats fruits and vegetables at meals or snacks; Beverage Choices; average of 2 items: My child drinks soda pop or sugar drinks (reverse coded) + My child drinks low fat milk at meals or snacks; Restriction and Reward; average of 2 items: Our family monitors eating of chips, cookies, and candy + Our family uses candy as a reward for good behavior (reverse coded). FDR: False Discovery Rate; FNPA: Family Nutrition and Physical Activity; FN: family-home nutrition.

### 3.3. Food Insecurity and Dietary Intake

We found no significant associations between food insecurity and dietary intake. We further examined the relationship between food insecurity and BMI in a subgroup of children with complete data (*n* = 76; data not shown). Within this subgroup, we found no association between food insecurity and BMI.

## 4. Discussion

This study offers a unique contribution to the literature by examining the relationship between the FN environment and obesity-related behaviors among rural elementary school-aged children. Obesity prevalence is higher among rural children [7,8,9,10,11], yet the causal pathways remain unclear. Our findings indicate that more favorable FN environment factors were associated with healthier dietary intakes, including higher consumption of fruits, vegetables, and dairy, and lower consumption of added sugar; eating habits that may help children to achieve and maintain a healthy BMI and prevent chronic disease [38].

Certain associations between FN and children’s dietary intake were as expected, such as the positive association between more frequent consumption of fruits and vegetables at meals or snacks and greater fruit and vegetable intakes among children. Likewise, children who more frequently drank low-fat milk at meals or snacks consumed more dairy, and children whose parents reported less frequent consumption of sugar-sweetened beverages consumed less added sugar.

Our results show that other FN factors were also associated with children’s intakes of fruits, vegetables, and added sugars. For example, less frequent use of microwave or ready-to-eat foods was associated with lower intake of added sugar and tended to be associated with higher intakes of vegetables. More frequent monitoring of chips, cookies, and candy consumption was also associated with higher vegetable intakes. Additionally, eating breakfast and family meals more often were both positively associated with fruit intake. Conversely, some FN factors that have previously been associated with dietary intake and/or weight status, such as watching TV while eating [21,25,46,47] and fast food consumption [15,25], were not associated with dietary intake among children in our study.

We found no associations between FN factors and intake of protein foods or whole grains. This finding is not surprising, given that the FNPA screening tool [12] does not include items specific to protein foods or whole grains. Protein intake in the US is typically adequate and, therefore, perhaps of less importance to the FN environment; however, most children do not meet current dietary recommendations for whole grains [38,48,49]. Whole grain consumption has been associated with multiple health outcomes, including healthier BMI [39,50]. Thus, future studies of FN environments may be strengthened by including assessment of the availability and accessibility of whole grain foods.

We observed no associations between family food insecurity and children’s dietary intakes or BMI. Previous research examining these associations among non-rural children is equivocal [28,29,30,31,32]. For example, Fram and colleagues found that greater levels of food insecurity were associated with poorer dietary intakes and lower levels of physical activity [31]. Conversely, Trapp and colleagues reported no difference in dietary intake among children from food-insecure households compared to those from food-secure households [32]. Differences in study findings may be partially explained by differences in sample populations and study methodology. For example, we used a 2-item screener to identify families at risk for food insecurity [40]. Although this instrument was previously found to be a valid identifier of households at risk for food insecurity among low-income families with young children, it does not allow for analysis based on different levels (e.g., household, adult, child) and categories (e.g., high, marginal, low) of food insecurity. Despite mixed findings on the association between food insecurity and dietary intake, other research indicates that food insecurity is associated with fewer opportunities for physical activity [51,52] and lack of access to healthy and affordable foods [53,54,55,56,57,58], factors that influence dietary and activity habits and may further influence ability to achieve and maintain healthy weight status. However, limited research has examined these associations in rural children. Larger studies including comprehensive measurement of food insecurity and dietary behaviors may be important for examining the complexities of this potential association.

The cross-sectional design of this study does not allow for causal inference. Other limitations include selection bias and self-reported data. To minimize bias in our results, we used validated survey instruments. We acknowledge that the relatively small number of families who enrolled in the larger GROW study, and the narrow geographic area in which the study was conducted, limit the generalizability of our findings. Another potential limitation was the use of a brief food frequency questionnaire (*i.e.*, food screener) to assess dietary intake. The 24-h multiple pass recall method, conducted over a 3-day period with parents as proxy reporters, has been suggested as the most accurate method to estimate total energy intake in children [59]. The BKFS instrument used in our study underestimates total energy intake; however, it has been shown to have good relative validity for assessing food group and added sugar intakes when compared against three 24-h recalls [36], and it is less burdensome for participants. We examined dietary intake as both absolute amounts of food groups (cups or g/day) and food group intakes standardized per 1000 kcals (4128 kJ) (*i.e.*, food density). Our results indicate that children in our sample did not meet recommended intake levels for vegetables, whole grains, or added sugars [38]. These findings are consistent with data from the 2003 to 2004 National Health and Nutrition Examination Survey (NHANES) [49] and other studies that have used the BKFS [36,60].

To our knowledge, this is the first study to examine the FNPA screening tool [12] in association with dietary intake. We found that some, but not all, FN factors were associated with dietary intake in a sample of rural children. Specifically, more favorable FN factors were associated with higher intakes of fruits, vegetables, and dairy, and lower intake of added sugar. Thus, our findings suggest that the FNPA screener captures multiple factors in the FN environment that support eating habits consistent with current dietary guidance for children [38,48]. Promoting healthy FN environments, such as the provision of vegetables, fruits, and low-fat dairy at meals and snacks; eating breakfast and family meals; and encouraging less frequent consumption of soda and ready-to-eat foods, may help to establish healthy dietary behaviors among rural children.

Future studies on the relationship between FNPA factors and childhood obesity would benefit from including direct measures of children’s diet and physical activity habits, as well as BMI, to clarify which FNPA components are central to promoting healthy weight-related behaviors during childhood. Longitudinal studies including larger, demographically diverse samples are recommended. Finally, future research examining associations between childhood obesity, food insecurity, and diet is warranted. Rural populations experience higher rates of obesity and food insecurity compared to other populations [26,27]; thus, examining these associations in rural settings is particularly important.

## 5. Conclusions

The family-home environment is a key setting for the development of healthy eating behaviors that may influence weight status later in life. Findings from our study suggest that more favorable FN environments are associated with healthier dietary intakes among rural elementary school-age children. To promote healthy eating habits in rural family-home environments, parents and caregivers may implement strategies including offering vegetables, fruits, and low-fat dairy at meals and as snacks, ensuring that children eat breakfast and meals together as a family, and limiting the availability of soda and ready-to-eat foods at home.
